# A novel immune-related lncRNA signature predicts the prognosis and immune landscape in ccRCC

**DOI:** 10.18632/aging.205633

**Published:** 2024-03-13

**Authors:** Longlong Dai, Daen Pan, Jiafei Jin, Wenhui Lv

**Affiliations:** 1 Department of Urology, Yongjia People’s Hospital, Wenzhou 325100, China

**Keywords:** immune, lncRNA, ccRCC, drugs

## Abstract

Background: As one of the most common tumors, the pathogenesis and progression of clear cell renal cell carcinoma (ccRCC) in the immune microenvironment are still unknown.

Methods: The differentially expressed immune-related lncRNA (DEirlncRNA) was screened through co-expression analysis and the limma package of R, which based on the ccRCC project of the TCGA database. Then, we designed the risk model by irlncRNA pairs. In RCC patients, we have compared the area under the curve, calculated the Akaike Information Criterion (AIC) value of the 5-year receiver operating characteristic curve, determined the cut-off point, and established the optimal model for distinguishing the high-risk group from the low-risk group. We used the model for immune system assessment, immune point detection and drug sensitivity analysis after verifying the feasibility of the above model through clinical features.

Results: In our study, 1541 irlncRNAs were included. 739 irlncRNAs were identified as DEirlncRNAs to construct irlncRNA pairs. Then, 38 candidate DEirlncRNA pairs were included in the best risk assessment model through improved LASSO regression analysis. As a result, we found that in addition to age and gender, T stage, M stage, N stage, grade and clinical stage are significantly related to risk. Moreover, univariate and multivariate Cox regression analysis results reveals that in addition to gender, age, grade, clinical stage and risk score are independent prognostic factors. The results show that patients in the high-risk group are positively correlated with tumor infiltrating immune cells when the above model is applied to the immune system. But they are negatively correlated with endothelial cells, macrophages M2, mast cell activation, and neutrophils. In addition, the risk model was positively correlated with overexpressed genes (CTLA, LAG3 and SETD2, P<0.05). Finally, risk models can also play as an important role in predicting the sensitivity of targeted drugs.

Conclusions: The new risk model may be a new method to predict the prognosis and immune status of ccRCC.

## INTRODUCTION

Kidney cancer is a malignant tumor with high invasiveness and poor prognosis that accounts for 4% of adult malignancies [1]. As the most common histologic subtype, clear cell renal cell carcinoma (ccRCC) originates from the epithelial cells of the proximal renal tubule [2]. Although, imaging technology could detect tumors at an early stage, one-third of patients still progress and even develop systemic metastases. Surgical operation is the gold standard for treatment of ccRCC, due to its resistance to chemotherapy and radiotherapy [3]. However, immune therapy has emerged as a promising strategy in cancer treatment during the past several decades [1]. A large number of evidences have been proved that ccRCC was associated with the abnormal expression of some immune-related genes, such as VHL, VEGF, PD-1 and m-TOR [2, 3]. According to past studies, anti-angiogenesis drugs and rapamycin drugs for ccRCC have become popular and changes the treatment of kidney cancer [4]. However, the treatment efficacy of these drugs was still not ideal and the patients eventually process and passed away. Therefore, understanding the pathogenesis and immune microenvironment of ccRCC was crucial for the treatment of renal cancer and the development of new drugs.

Long noncoding RNAs (lncRNAs) belongs to non-protein-coding RNA family and are more than 200 nucleotides in length [5]. Recently, more and more clues indicated that lncRNAs play an important role in the regulation of the immune system [6]. As an intergene lncRNA, the expression of NeST in T cells of SJL/J mice containing SJL strain was significantly higher than that of T cells of SJL/J mice without SJL strain. And NeST expression could produce more IFN-γ in activated CD8^+^ T cells [7]. Moreover, it is proved that lncRNA EPS is down-regulated in macrophages and DCs after the innate immune system been activated [8]. Similarly, lncRNA lnc13 also functions as a repressor of inflammatory responses by activated TLR4 in celiac disease [9]. In addition, Hong et al. found that 12 immune-related lncRNA (irlncRNA) signatures could better predict the prognosis of patient with hepatocellular carcinoma (HCC) and immune landscape of HCC [10]. However, there are few studies on immune-related lncRNAs in predicting prognosis and immunological background of renal cancer. Therefore, further exploration and study of immune-related lncRNAs may be new checkpoints that will be of great benefit to the diagnosis and treatment of kidney cancer. In our study, we constructed the irlncRNAs risk model to predict the prognosis and immune landscape of RCC using the data from TCGA database. Moreover, tumor-immune infiltration cell and targeted-drug sensitivity also were analyzed in our study.

## MATERIALS AND METHODS

### Data acquired and processing

Transcriptome profiling data and clinical character of ccRCC patients were acquired from The Cancer Genome Atlas database (TCGA, https://portal.gdc.cancer.gov/) and the above downloaded data from TCGA database were collated and ID converted with Strawberry Perl (version 5.30.0.1-64bit, https://strawberryperl.com/). The gene transfer format (GTF) file from Ensembl was used to annotate the international standard gene names. Immune-related gene list was obtained from Immport database (https://www.immport.org/) to screen the immune-related genes.

### Screening the differentially expressed immune-related lncRNAs

The immune-related lncRNAs were handled by the R language software (version 3.5.0, https://www.r-project.org/). The limma package in the Bioconductor package (http://www.bioconductor.org/) was used to distinguish lncRNA expression analysis. And the thresholds for screening differentially expressed immune-related lncRNAs (DEirlncRNAs) were as follows: *P<0.05* and *[log2 FC]≥1*. The above results are represented by volcano map and hierarchical clustering heat map.

### Constructing the immune-related lncRNA pair

In order to integrate transcriptome data and clinical data better, DEirlncRNA pairs were constructed by iteration loop and a 0-or-1 matrix [10]. Next, immune-related lncRNA pairs were combined with survival data of patient with ccRCC to screen out DEirlncRNA pairs with prognostic effect.

### Constructing the risk model and getting the riskscore

Least absolute contraction and selection operator (LASSO) regression analysis and lambda spectra are used to screen DEirlncRNA pairs in order to prevent overfitting when constructing a prognostic risk model. In our research, Sveen modified the LASSO regression analysis as follows [11]: Run 1000 cycles, and set 1000 random stimuli in each cycle. Next step, recording the frequency of each pair of Lasso regression models that are repeated 1000 times, and select the pairs with a frequency of more than 100 times to perform Cox proportional hazard regression analysis and build the model. Then the AUC values of these models were calculated. When the AUC value reaches the maximum value, it indicates that the model is the best candidate model. Next, apply the time-dependent receiver operating characteristic curve (ROC) to evaluate the accuracy of the R package (survivalROC) risk model. In this study, the risk score is obtained by the following formula: $\text{RiskScore}=\sum_{i-1}^{k}\beta iSi$. Finally, calculate the Akaike Information Criterion (AIC) value of the 5-year ROC curve to obtain the cut-off value of the high and low risk groups.

### Validating the risk model

Kaplan-Meier survival curve was performed in order to compare the overall survival (OS) in the high and low risk groups. And risk score distribution and survival status were drawn down.

In order to verify the clinical value of the risk model, the chi-square test was used to correlate the model with clinicopathological characteristics. Moreover, the differences in risk scores between groups of these clinicopathological characteristics were analyzed by Wilcoxon signed rank test. Univariate and multivariate Cox regression analysis were performed to assess the relationship between risk score and independent clinic-pathological risk factors which contain age, gender, grade and stage. At the same time, the ROC curve is used to show the predictive potential of the risk model. The R package used to handle the above operations, including survminer, glmnet, survivalROC, pHeatmap and ggupbr. *P<0.01* was considered as meaningful.

### The relationship between tumor-infiltrating immune cells and risk assessment model

The following methods (such as XCELL [12], TIMER [13], QUANTISEQ [14], MCPCOUNTER [15], EPIC [16], CIBERSORT-ABS [17] and CIBERSORT [18]) were applied to explore the relationship between the risk scores and tumor-infiltrating immune cell. The Wilcoxon signed-rank test was used to analyze the differences in the content of immune infiltrated cells explored by the above methods between the high-risk and low-risk groups. Besides, the relationship between the risk scores and immune infiltrated cells was displayed by spearman correlation analysis. And a lollipop diagram was used to display the above result. The operation was performed by ggplot2 package for R.

### Immunosuppressed molecules valid and drug sensitivity analysis

In our study, the immunosuppressive molecule was visualized by the ggstatsplot package. And the violin plot was used to detect the expression of the above ICIs-related genes. According to the EUA guideline, axitinib, bosutinib, imatinib, sunitinib, sorafenib, pazopanib and temsirolimus were recommended as a first-line or second-line clinical targeted drugs for ccRCC [19]. The IC_50_ of the above antitumor drugs was calculated to assess the clinical application of the risk model in ccRCC. The operation was performed by pRRophetic and ggplot2 package.

### Availability of data and materials

Supplemental information can be found online.

## RESULTS

### Get DEirlncRNAs

**Figure 1:**
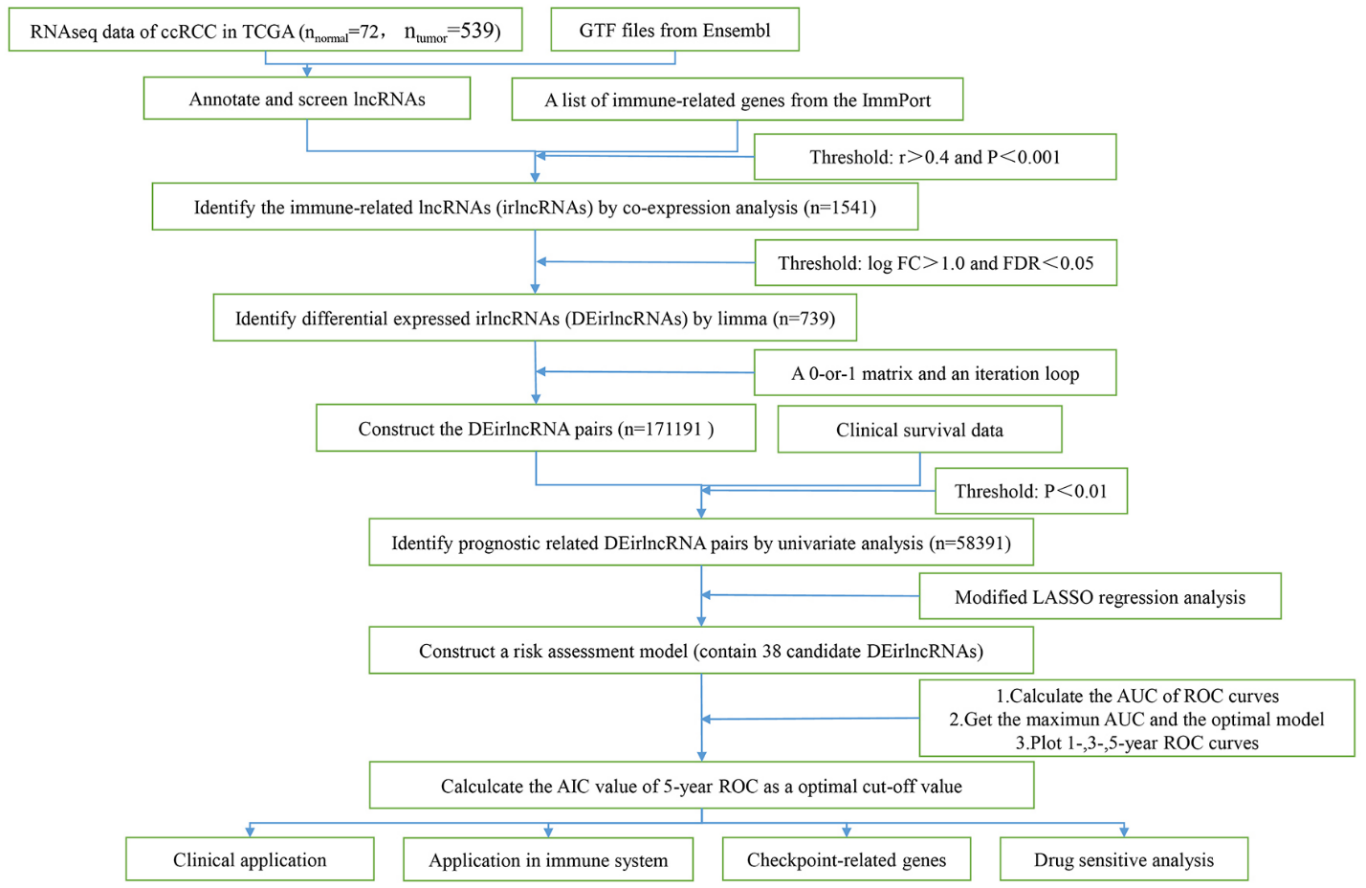
The flow chart.

**Figure 2:**
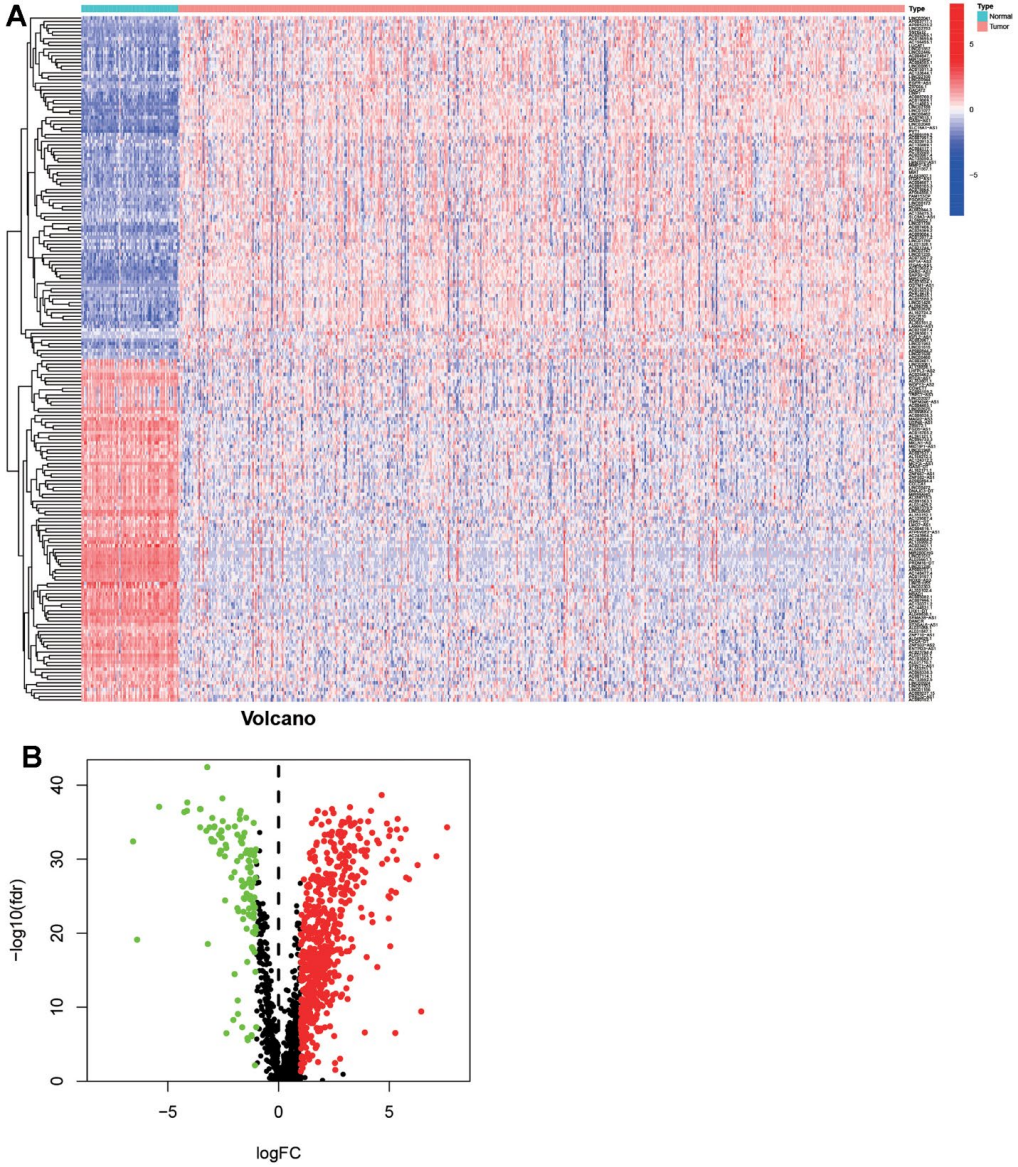
**Differentially expressed immune-related lncRNAs (DEirlncRNAs) were screened and verified.** The hierarchical clustering heat map which contained top-100 DEirlncRNAs (**A**) and volcano (**B**) map were shown.

As shown in Figure 1, 72 normal samples and 539 ccRCC samples were acquired from the ccRCC project of the TCGA database. Then the GTF file was used to annotate the international standard gene names, and 14086 lncRNAs and 19604 mRNAs were obtained. 1541 irlncRNAs were screened out through further analysis by co-expression analysis. 738 irlncRNAs were identified as DEirlncRNAs, including 376 up-regulated irlncRNAs and 362 down-regulated irlncRNAs by performing R limma package. As shown in Figure 2, DEirlncRNAs were displayed by hierarchical clustering heat map (contain top-100 DEirlncRNAs) and volcano map.

### Construct the DEirlncRNAs pairs and a risk assessment model

**Figure 3:**
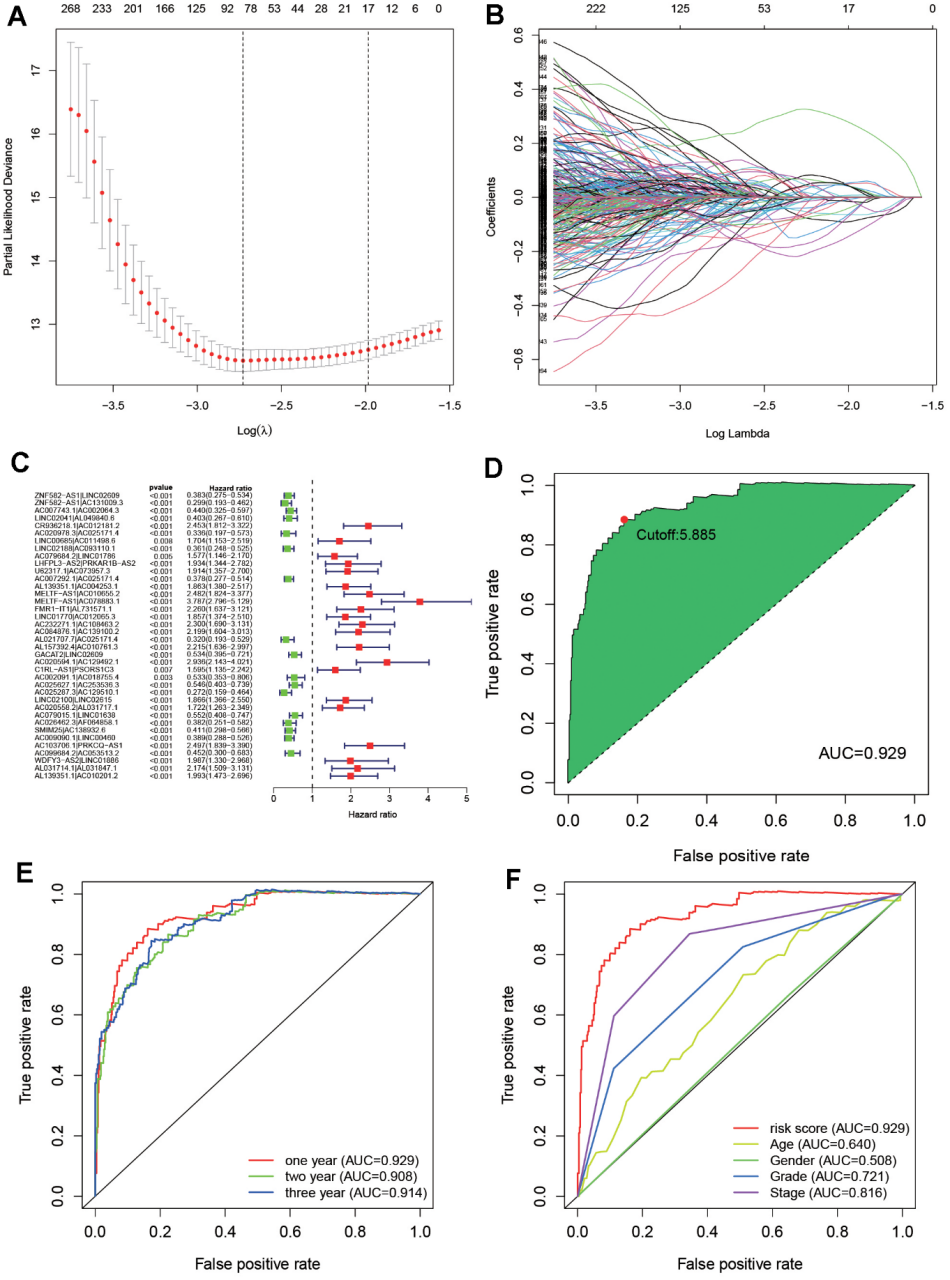
**Construct an optimal risk assessment model.** (**A**) Cross-validation for tuning parameter selection by the modified least absolute shrinkage and selection operator analysis (LASSO). (**B**) LASSO coefficient profiles of 38 prognostic immune-related genes. (**C**) The univariable Cox model result was shown by forest plot. (**D**) Calculate the maximum AIC value generated by ROC curves of 38 DEirlncRNAs and get the optimal cut-off value. (**E**) The 1-, 3-, 5-year ROC of the optimal model revealed that all AUC values were over 0.90. (**F**) A comparison of 5-year ROC curves with other common clinical characteristics showed the superiority of the riskScore.

171191 DEirlncRNA pairs were constructed through a 0- or -1 matrix and an iterative loop after the effective DEirlncRNA was screened out. Through univariate analysis, 58391 DEirlncRNA pairs related to prognosis were mined (*P<0.01*) but which has to combing with DEirlncRNA pairs and survival data. Then, by modified LASSO regression analysis, 38 candidate DEirlncRNA pairs were included in a risk assessment model (Figure 3A–3C and Table 1).

**Table 1:** The risk model including 38 lncRNA pairs.

**ID**	**HR**	**HR.95L**	**HR.95H**	**p-value**
ZNF582-AS1|LINC02609	0.3829	0.2747	0.5339	0.0000
ZNF582-AS1|AC131009.3	0.2989	0.1934	0.4619	0.0000
AC007743.1|AC002064.3	0.4404	0.3249	0.5968	0.0000
LINC02041|AL049840.6	0.4033	0.2667	0.6098	0.0000
CR936218.1|AC012181.2	2.4533	1.8120	3.3217	0.0000
AC020978.3|AC025171.4	0.3358	0.1967	0.5732	0.0001
LINC00685|AC011498.6	1.7040	1.1525	2.5192	0.0075
LINC02188|AC093110.1	0.3608	0.2478	0.5251	0.0000
AC079684.2|LINC01786	1.5774	1.1465	2.1702	0.0051
LHFPL3-AS2|PRKAR1B-AS2	1.9339	1.3445	2.7817	0.0004
U62317.1|AC073957.3	1.9140	1.3569	2.6996	0.0002
AC007292.1|AC025171.4	0.3776	0.2772	0.5142	0.0000
AL139351.1|AC004253.1	1.8634	1.3797	2.5169	0.0000
MELTF-AS1|AC010655.2	2.4818	1.8238	3.3772	0.0000
MELTF-AS1|AC078883.1	3.7868	2.7960	5.1286	0.0000
FMR1-IT1|AL731571.1	2.2602	1.6369	3.1208	0.0000
LINC01770|AC012065.3	1.8569	1.3736	2.5104	0.0001
AC232271.1|AC108463.2	2.3003	1.6898	3.1313	0.0000
AC084876.1|AC139100.2	2.1986	1.6043	3.0131	0.0000
AL021707.7|AC025171.4	0.3196	0.1933	0.5285	0.0000
AL157392.4|AC010761.3	2.2146	1.6363	2.9974	0.0000
GACAT2|LINC02609	0.5340	0.3953	0.7214	0.0000
AC020594.1|AC129492.1	2.9356	2.1431	4.0211	0.0000
C1RL-AS1|PSORS1C3	1.5952	1.1350	2.2419	0.0072
AC002091.1|AC018755.4	0.5334	0.3530	0.8060	0.0028
AC025627.1|AC253536.3	0.5458	0.4033	0.7388	0.0001
AC025287.3|AC129510.1	0.2720	0.1594	0.4640	0.0000
LINC02100|LINC02615	1.8664	1.3663	2.5495	0.0001
AC020558.2|AL031717.1	1.7225	1.2632	2.3488	0.0006
AC079015.1|LINC01638	0.5524	0.4085	0.7470	0.0001
AC026462.3|AF064858.1	0.3821	0.2510	0.5817	0.0000
SMIM25|AC138932.6	0.4105	0.2977	0.5661	0.0000
AC009090.1|LINC00460	0.3894	0.2883	0.5260	0.0000
AC103706.1|PRKCQ-AS1	2.4969	1.8391	3.3900	0.0000
AC099684.2|AC053513.2	0.4521	0.2995	0.6825	0.0002
WDFY3-AS2|LINC01886	1.9869	1.3302	2.9679	0.0008
AL031714.1|AL031847.1	2.1737	1.5089	3.1313	0.0000
AL139351.1|AC010201.2	1.9930	1.4732	2.6961	0.0000

In order to obtain an ideal risk assessment model, the area under the curve (AUC) of each ROC curve is calculated. As shown in Figure 3D, when the cut-off value is 2.895, AUC reaches the maximum value (AUC=0.942). Then, in order to verify the above model, ROC curves of 1, 3, and 5 years were drawn (Figure 3E), and all of their AUC values were greater than 0.90. In addition, the relationship between the 5-year ROC curve and other clinical characteristics was also plotted (Figure 3F). According to the Akaike information criterion, the cut-off value on the 5-year ROC curve is regarded as the maximum inflection point.

### Clinical application of risk assessment model

**Figure 4:**
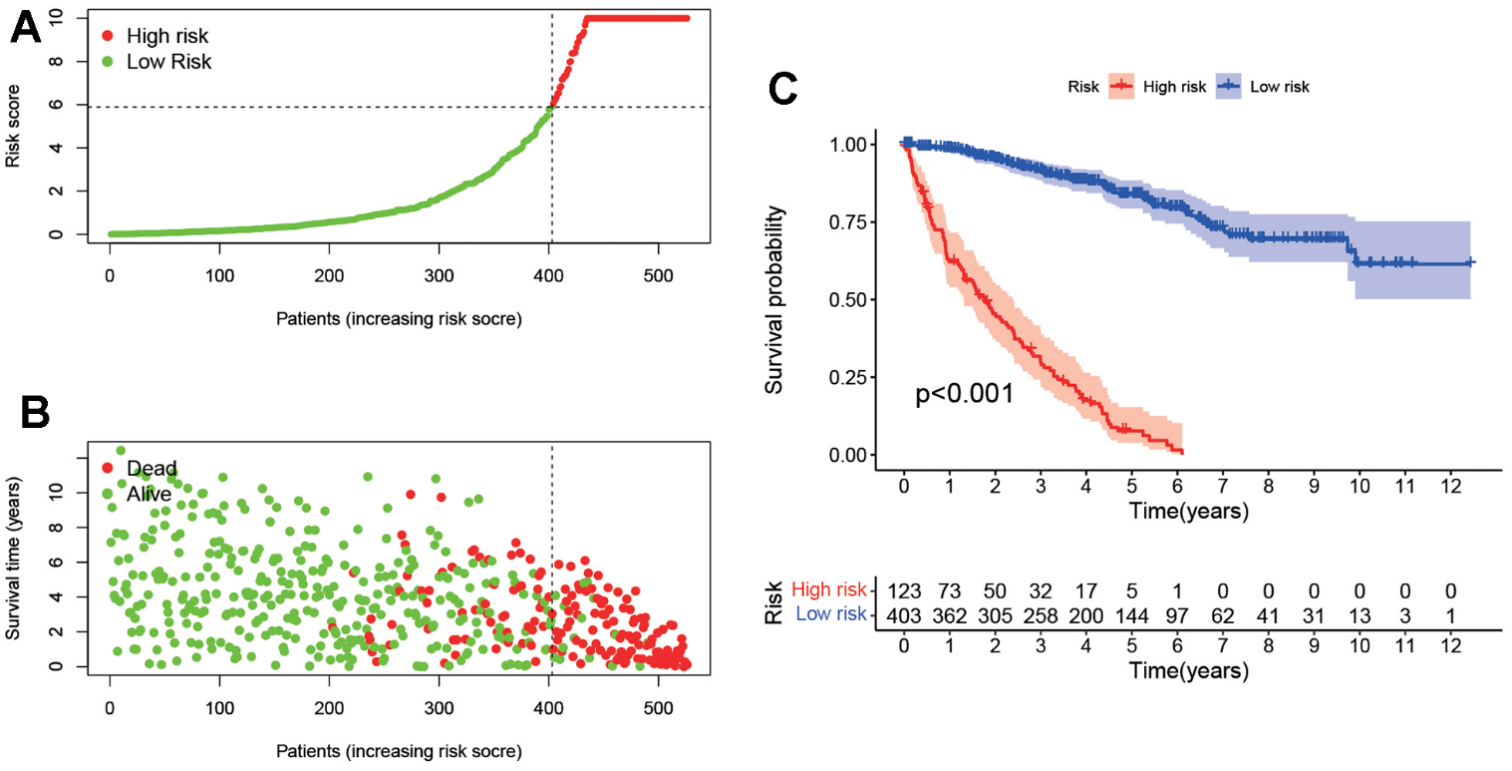
**Survival analysis according to the risk assessment model.** (**A**) The distribution of risk scores. (**B**) The relationship between risk scores and survival times. (**C**) Kaplan-Meier survival curves suggested that the patients in low-risk group have a better prognosis.

**Figure 5:**
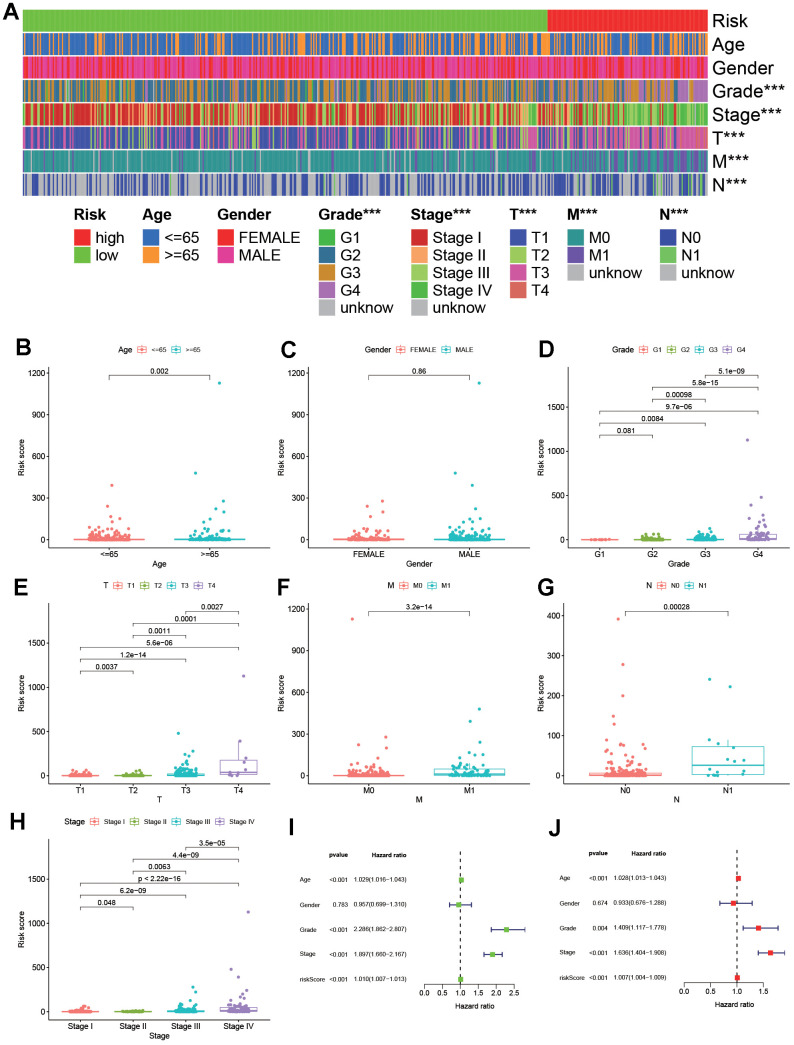
**Verifying the risk assessment model in clinical application.** (**A**) A strip chart. (**B**–**H**) The scatter diagrams displayed the relationship between clinical characteristics and rick scores. Besides, the univariate (**I**) and multivariate (**J**) Cox hazard ratio analysis revealed that age, grade, stage and riskScore were presented as independent prognostic predictor.

As shown in Figure 4A, 4B, according to the effective cut-off value, the 526 ccRCC patients in the TCGA database were divided into two groups: a high-risk group (123 cases) and a low-risk group (403 cases). The results showed that the prognosis of ccRCC patients in the low-risk group was significantly better than that of the high-risk group (*P<0.001*, Figure 4C). Moreover, heat maps and block diagrams (Figure 5A–5H) show that T stage, M stage, N stage, grade and clinical stage are significantly related to risk, but not related to age and gender by comparing the relationship between risk and clinical characteristics. And through univariate and multivariate Cox regression analysis (Figure 5I, 5J) further analysis, the results show that age, grade, clinical stage and risk score as independent prognostic factors, excluding gender.

### Application of risk assessment model in immune system

**Figure 6:**
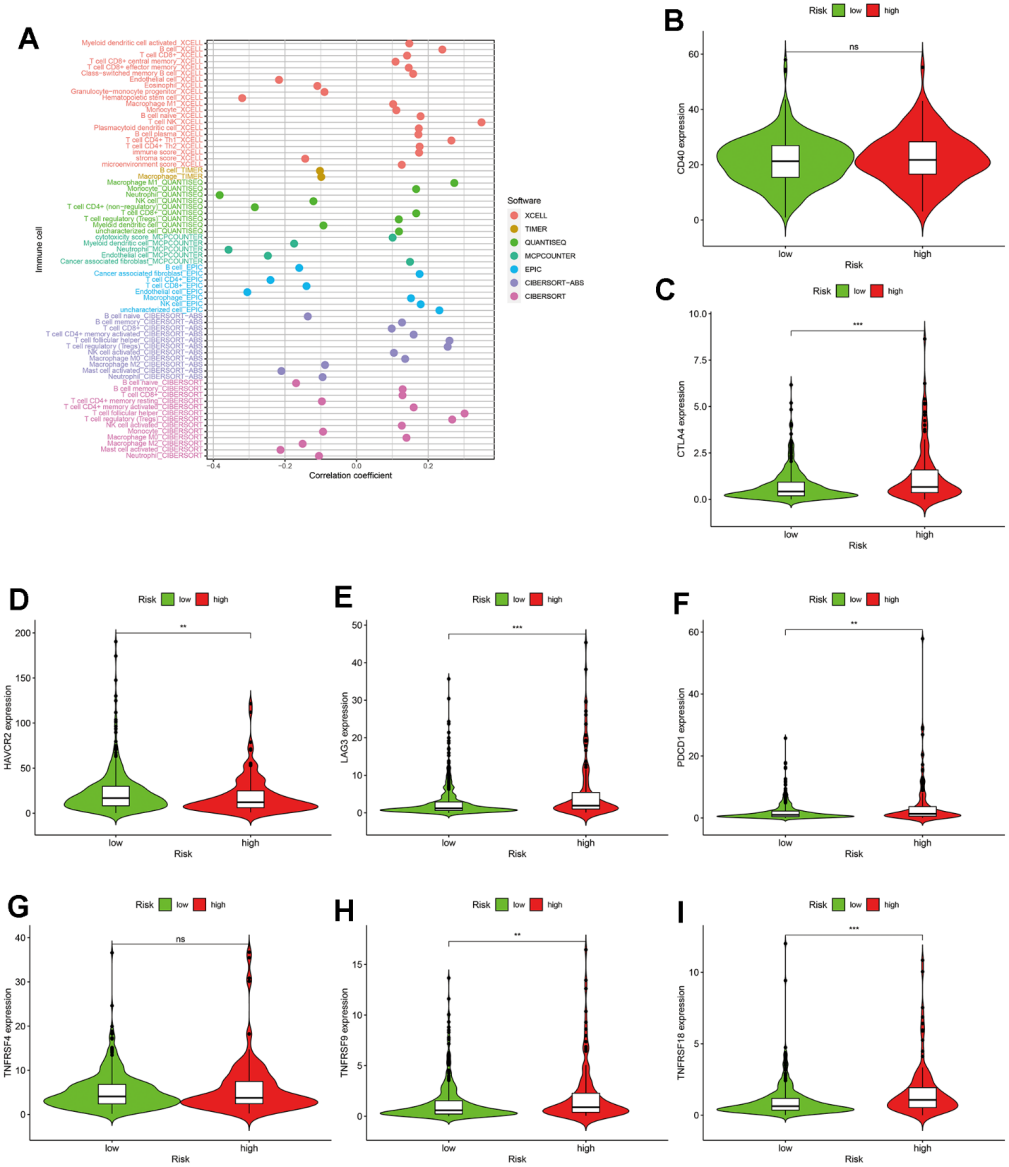
**The application of the risk assessment model in tumor-infiltrating cells and immunosuppressed molecules.** (**A**) The lollipop diagram. (**B**–**I**) The relationship between risk scores and immunosuppressed molecules.

To explore the relationship between the immune system and the risk assessment model, recognized methods were used, including XCELL, TIMER, QUANTISEQ, MCPCOUNTER, EPIC, CIBERSORT-ABS, and CIBERSORT. As a result, the lollipop chart (Figure 6A) shows that patients in the high-risk group are positively correlated with tumor-infiltrating immune cells (such as B cell memory, cancer-related fibroblasts, macrophages M0, macrophages M1, and T cell CD4+ memory) more activated T cell follicular helper cells and T cell regulators (Tregs), and they are negatively correlated with endothelial cells, macrophages M2, mast cell activation and neutrophils by spearman correlation analysis.

### ICIs-related genes valid and drug sensitive analysis

**Figure 7:**
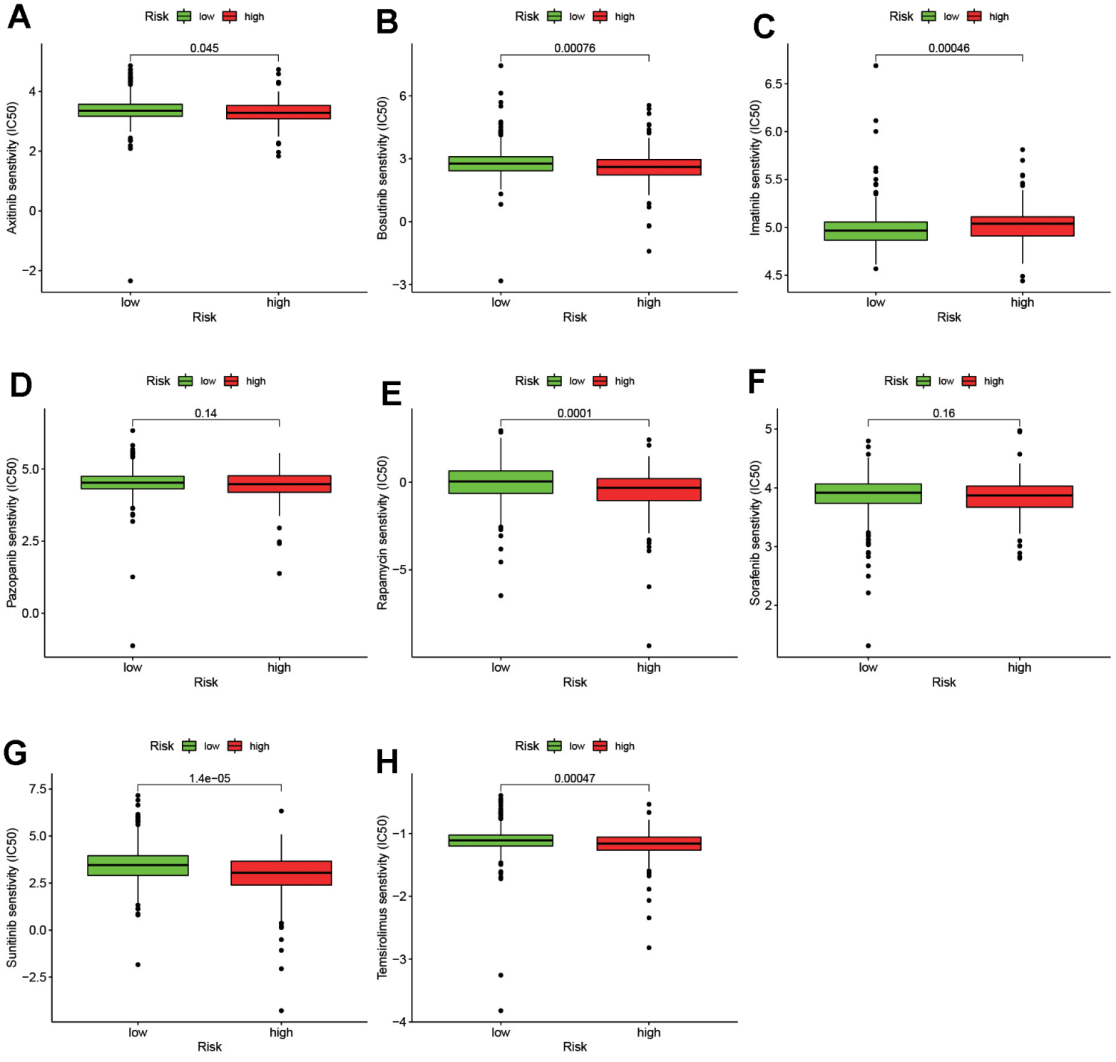
The relationship between the risk assessment model and common targeted drugs, including axitinib (**A**), bosutinib (**B**), imatinib (**C**), pazopanib (**D**), rapamycin (**E**), sorafenib (**F**), sunitinib (**G**), temsirolimus (**H**).

Currently, immune checkpoint inhibitors have been widely used clinically [20, 21]. Therefore, it is necessary to improve the relationship between immunosuppressive molecules and risk models. The results (shown in Figure 6B–6I) indicated that CTLA4 (*P<0.001*), LAG3 (*P<0.001*), PDCD1 (*P<0.01*), TNFRSF9 (*P<0.01*) and TNFRSF18 (*P<0.001*) were significantly positively correlated with high risk scores, while HAVCR2 (*P<0.01*) was obviously negatively correlated with high risk scores. There was no statistically significant difference with CD40 and TNFRSF4 (*P>0.05*). Moreover, we tried to clarify the connection between the efficacy of common targeted drugs and risk models. The result is shown in Figure 7. The higher IC_50_ of targeted drugs is associated with a high risk score, such as imatinib (*P=0.000*). In contrast, the lower IC_50_ of targeted drugs is associated with high risk scores, such as axitinib (*P=0.045*), bosutinib (*P=0.001*), sunitinib (*P=0.000*), and sirolimus (*P=0.000*). The latter two (pazopanib and sorafenib) had no significant difference (*P>0.05*). The above results indicate that the risk model plays an important role in predicting the sensitivity of targeted drugs.

## DISCUSSION

As one of the most aggressive tumors, the efficacy of RCC remains unsatisfactory, especially in advanced renal cancer [22]. Therefore, it is important to understand the mechanism of RCC and to search for new targets. Currently, there was many evidence identified that a number of genes are associated with the development of kidney cancer. For example, as a famous gene in ccRCC, mutation of VHL can activate the HIF pathway, which results in dysfunction of a number of factors that regulate proliferation, migration, invasion and apoptosis [23]. Besides, the mutations of BAP1, PBRM1 and SETD2 also increase the risk of ccRCC [24]. Based on the above mechanisms, immunotherapy has been widely used in the treatment of advanced renal cancer [25].

In recent years, many studies have proved that lncRNAs play as key role in regulating the expression of immune genes and may be important prognostic markers of tumors [26, 27]. For example, Xu found that lncRNASATB2-AS1 is down-regulated in colorectal cancer, which might be a dependent prognostic biomarker for CRC. Further functional experiments showed that lncRNA SATB2-AS1 can regulate the expression of TH1 chemokines and immune cell density in CRC [28]. As a long non-coding RNA that interacts with NF-κB, lncRNA NKILA inhibits the expression of NF-κB, thereby regulating the sensitivity of T cells to activation-induced cell death [29]. In addition, lncRNA cox2 inhibits the immune escape of HCC cells and tumor progression by inhibiting the polarization of macrophages from M1 to M2 [30].

However, the specificity and sensitivity of a single lncRNA in tumor prediction is still not satisfactory. Therefore, the construction of multiple-lncRNA signature to improve the diagnosis and prognosis of cancer has become a new method [30–32]. At the present study, the raw data of lncRNAs was obtained from TCGA database. First, 739 DEirlncRNAs were screened out by a differential co-expression analysis and the limma package for R. Then 171191 DEirlncRNA pairs were constructed by a 0-or-1 matrix and an iteration loop. Second, to improve the accuracy and efficacy of prediction on risk, the univariate analysis combined with a modified LASSO regression analysis was performed to valid DEirlncRNA pairs. Third, to get the optimal model, each AUC value of ROC and the AIC value of each point on the AUC were calculated. Based on the above result, the optimal cut-off point was used to differentiate the high or low risk-group among patients with ccRCC. Fourth, the novel risk model was assessed by the following situation, including survival, clinical pathological characteristics, tumor-infiltrating immune cells, ICIs and targeted-drug sensitive. Among of them, ICIs are monoclonal antibodies that block suppressor molecules on T cells or their ligands to activate the tumor immune response. We collected important immunosuppressive molecules associated with ccRCC in recent years, such as PD-1, CTLA-4, TIM3, LAG3 and et al. [33]. As a result, CTLA4, LAG3, PDCD1, TNFRSF9 and TNFRSF18 were significantly positively correlated with high risk scores, while HAVCR2 was obviously negatively correlated with high risk scores. As we know, ccRCC is not sensitive to chemotherapy and radiation therapy, but some targeted drugs have a good therapeutic effect on kidney cancer [34]. Therefore, we also analyzed the correlation between this model and chemotherapy drugs and targeted drugs for renal cancer. In the present study, the higher IC_50_ of targeted drugs is associated with a high risk score, such as imatinib. In contrast, the lower IC_50_ of targeted drugs is associated with high risk scores, such as axitinib, bosutinib, sunitinib, and sirolimus. These results may provide a theoretical basis for clinicians to choose appropriate drugs for the treatment of ccRCC in the future.

There were some studies about ccRCC and irlncRNA signatures. For example, Sun et al. filtered out 5 irlncRNAs related to prognosis from the TCGA database and constructed prognostic risk characteristics. Results reveals that the above-mentioned irlncRNA characteristics was not only independent prognostic factors of ccRCC, but also related to clinical characteristics [35]. Similarly, Jiang et al. constructed three irlncRNA features as the prognostic risk model of ccRCC, and obtained similar results to sun [36]. If we compared with the above studies, our research has the following advantages: (1) By constructing immune lncRNA pairs, we only need to detect high or low expression pairs, avoiding redundant algorithms and reducing heterogeneity; (2) Our model has clinical applicability, which can distinguish between high and low risks of clinical cases; (3). Our model can also be used to detect ICIs-related genes and drug sensitivity analysis.

Although some positive results have been achieved, the current research has some shortcomings. One hand, the risk model was constructed by a public database (TCGA). The predictive value of the risk model needs further verification in randomized controlled trials. On the other hand, as our results were all derived from bioinformatics analysis, the expression and functional role of DEirlncRNAs obtained above in ccRCC should be verified in the further.

## CONCLUSIONS

In a word, the new model constructed in this study helpful in predicting the prognosis of patients with ccRCC, and also supported in anti-tumor immunotherapy. However, it requires proactive verification.
